# Effects of Composition and Baking Temperature on the Properties of Low‐Protein Cookies

**DOI:** 10.1002/fsn3.72069

**Published:** 2026-06-30

**Authors:** Cuiping Shi, Ye Zi, Zhenfeng Liu, Wei Cai, Jian Zhong

**Affiliations:** ^1^ Shanghai Key Laboratory of Pediatric Gastroenterology and Nutrition Xinhua Hospital, Shanghai Jiao Tong University School of Medicine Shanghai China; ^2^ Department of Clinical Nutrition, College of Health Science and Technology Shanghai Jiao Tong University School of Medicine Shanghai China; ^3^ Medical Food Laboratory Shanghai Institute for Pediatric Research Shanghai China; ^4^ Shanghai Pharma Qingchunbao‐Xinhua Hospital Precision Nutrition Research Center Chiatai Qingchunbao Pharmaceutical Group Limited Zhejiang Hangzhou China

**Keywords:** bakery food, composition, gas chromatography‐ion mobility spectrometry, heat processing, sensory, texture

## Abstract

Low‐protein foods are important medical foods for the dietary management of chronic kidney diseases and some inherited metabolic disorders. This work focused on fabricating low‐protein cookies (LPCs) from low‐protein steamed bun flour via baking and elucidating the influence of both formulation variables and baking temperature on their physicochemical properties through single‐factor analysis. The formulation components and baking temperatures (100°C–200°C) were important for preparing LPCs. In particular, fish oil and corn oil synergically improved the textural properties of both the doughs (hardness of 146.0 N and fracturability of 146.0 N) and the LPCs (hardness of 44.7 N and fracturability of 44.0 N). Moderate baking temperatures (120°C–160°C) were necessary to produce LPCs with good quality. The hardness (4.4–27.8 N) and fracturability (4.4–26.2 N) increased (100°C–160°C), remained unchanged (160°C–170°C), and then decreased (170°C–200°C) with the increasing baking temperatures. At an accelerated storage at 57°C, all LPCs prepared at different baking temperatures showed a significant decrease in redness‐greenness. Furthermore, the textural and sensory stability were dependent on the baking temperatures and storage days. The in vitro free fatty acid released percentages increased (8.6%–18.9% after 2 h in the small intestinal phase) with the increasing baking temperature. The LPC with the baking temperature of 160°C had a protein content of 0.27 ± 0.01 g/100 g. This work provided useful information to understand the effects of composition and baking temperature on the properties of low‐protein foods.

## Graphical abstract

This work focused on fabricating low‐protein cookies (LPCs) from low‐protein steamed bun flour via baking and elucidating the influence of both formulation variables and baking temperature on their physicochemical properties through single‐factor analysis. Moderate baking temperatures (120°C–160°C) were necessary to produce LPCs with good quality. Baking temperatures significantly affected the textural properties, textural and sensory stability, and in vitro free fatty acid released percentages of LPCs.

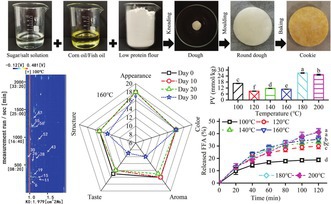

## 1 Introduction

Low‐protein foods (LPFs) are dietary foods with lower protein content than regular foods. They are indispensable for patients with certain special diseases, such as inherited amino acid metabolism disorders (Handoom et al. 2018), inherited organic acid metabolism disorders (Garcia‐Arenas et al. 2023), and chronic kidney diseases (Zhang et al. 2023). Moreover, LPFs represent a lifelong dietary nutritional requirement for these patients (Garcia‐Arenas et al. 2023; Ziadlou and MacDonald 2026). Therefore, the development of LPFs has attracted considerable attention from both the medical and food science communities.

LPFs have been studied over the past two decades. Four low‐phenylalanine pasta formulations were developed using wheat flour, corn starch, pectin, and carboxymethylcellulose (Yaseen and Shouk 2011). The total protein and phenylalanine contents in the low‐phenylalanine pasta reached 2.94–5.76 and 0.12–0.23 g/100 g dry sample, respectively, which were lower than those in regular pasta (9.76 and 0.38 g/100 g dry sample, respectively). Five low‐phenylalanine bread formulations were prepared using corn starch, sweet sprinkles, cassava flour, oil, refined sugar, and salt (Scortegagna et al. 2020). The total protein and phenylalanine contents reached 1.40–1.43 and 0.013–0.031 g/100 g bread, respectively. The effects of the type and amount of modified corn starches on the properties of low‐protein biscuits were also studied (Azaripour and Abbasi 2020). The total protein and phenylalanine contents reached 1.27 and 0.05 g/100 g biscuit, respectively, with 30% pregelatinized and 70% cross‐linked starch. The protein contents of these foods were all higher than 1.0 g/100 g. Further reduction in protein content should be pursued whenever possible. However, proteins serve dual roles as nutrients and structural building blocks in foods, and low protein content makes it difficult to produce LPFs with ideal structure, texture, and product stability. Therefore, it is important to develop more LPFs and to explore the effects of preparation factors on their properties.

Cookies are popular dry‐baked foods characterized by relatively high fat and sugar content (Tiwari et al. 2023). They are typically prepared using flour, oil, sugar, salt, and water (Boukid and Rosentrater 2024). Corn oil is a widely used vegetable oil around the world (Boukid and Rosentrater 2024). Fish oil is rich in omega‐3 polyunsaturated fatty acids and offers various health‐promoting benefits (J. Xu et al. 2024). Furthermore, baking temperature has a significant impact on the properties of oils in food products (Goh et al. 2019). Dough thickness and baking temperature have been shown to influence the nutrient composition of cookies made from wheat‐white yam flour (Pc et al. 2025). Nevertheless, no formulation for low‐protein cookies (LPCs) has been reported to date. Therefore, it is of great interest to develop LPCs and to investigate the effects of ingredients and processing factors on their properties.

This work aimed to develop LPCs with low protein contents (≤ 1.0 g/100 g) and to analyze the effects of preparation factors (ingredient addition parameters and baking temperatures) on the properties of LPCs, including textural properties, volatile organic compounds (VOCs), accelerated stability, sensory properties, and in vitro digestion behaviors. Finally, the nutritional components of the LPC baked at 160°C were determined to confirm its low protein content. The results will help elucidate the effects of preparation factors on the properties of LPFs. Moreover, this work will provide LPC formulations that allow patients on protein‐restricted diets to enjoy the pleasures of a normal diet.

## 2 Materials and Methods

### 2.1 Raw Materials

Low‐protein steamed bun flour (Aishushu, 0.47 g protein and 88.8 g carbohydrate per 100 g flour) was purchased from Shanghai Zhunshen Food Dongtai Co. Ltd. (Dongtai City, Jiangsu Province, China). Corn oil (Arawana, pressed from non‐genetically modified corn germ, 100.0 g fat per 100 g product) was purchased from Shanghai Kerry Food Industry Co. Ltd. (Shanghai, China). Fish oil (DHA + EPA ≥ 70%) was purchased from Xi'an Qianyecao Biological Technology Co. Ltd. (Xi'an City, Shaanxi Province, China). White granulated sugar (first grade, Yutang, purity ≥ 99.8%) was purchased from Dafeng Yinmore Sugar Industry Co. Ltd. (Yancheng, Jiangsu, China). Refined edible salt (Zhongyan, nutritional information: 0 g protein, 0 g fat, 0 g carbohydrate, 38,962 mg sodium, and 2.625 mg iodine per 100 g product) was purchased from Zhongyan Shanghai Salt Industry Co. Ltd. (Shanghai, China). Drinking water was purchased from China Resources Yibao Beverage (Yixing) Co. Ltd. (Yixing, Jiangsu, China).

### 2.2 Preparation of LPCs

**FIGURE 1:**
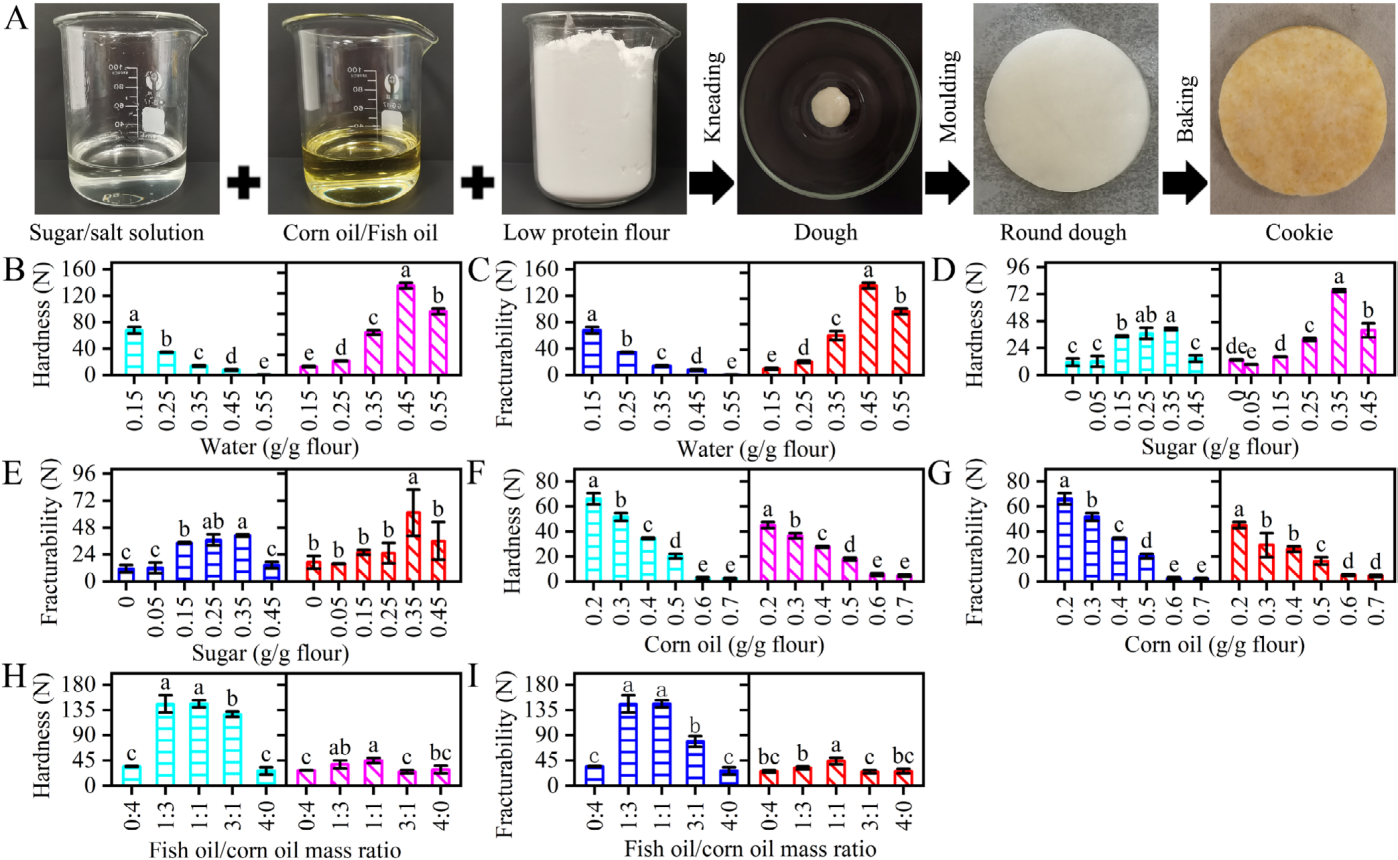
Effects of component parameters on the textural properties of the low‐protein cookies (LPCs). (A): Preparation process. (B, C): Effect of water addition contents on the hardness (B) and fracturability (C) of the doughs (Left parts) and LPCs (Right parts). (D, E): Effect of white granulated sugar addition contents on the hardness (D) and fracturability (E) of the doughs (Left parts) and LPCs (Right parts). (F, G): Effect of corn oil addition contents on the hardness (F) and fracturability (G) of the doughs (Left parts) and LPCs (Right parts). (H, I): Effect of fish oil/corn oil mass ratios on the hardness (H) and fracturability (I) of the doughs (Left parts) and LPCs (Right parts). The columns and error bars indicate the average values and standard deviation (*n* = 3). Different letters on the columns in each image indicate significant differences (*p* < 0.05) using one‐way ANOVA and Duncan's test.

The LPCs were prepared using low‐protein steamed bun flour as the main raw material via a simple baking method (Zi et al. 2026). As shown in Figure 1A, low‐protein steamed bun flour (20 g), water (0.25 g/g flour), corn oil (0.4 g/g flour), a fish oil‐to‐corn oil mass ratio of 0:4 (indicating no fish oil in the formulation), white granulated sugar (0.15 g/g flour), and refined salt (0.1 g) were mixed and kneaded to form a dough. Then, 10 g of the dough was molded into a round shape (4 cm in diameter and 6 mm in thickness). The round dough pieces were placed in a preheated electric oven (PT3520W‐G, Midea, Foshan, Guangdong, China) at 160°C. After baking for 12.5 min, the samples were flipped over and baked for another 12.5 min. Finally, the obtained cookies were taken out and cooled for 3 min at room temperature. The doughs and LPCs were photographed using a digital camera.

The effects of component addition parameters and baking temperatures were studied by varying one of the above parameters at a time. The following parameters were examined: water addition content (0.15, 0.25, 0.35, 0.45, and 0.55 g/g flour), white granulated sugar addition content (0.00, 0.05, 0.15, 0.25, 0.35, and 0.45 g/g flour), corn oil addition content (0.2, 0.3, 0.4, 0.5, 0.6, and 0.7 g/g flour), fish oil‐to‐corn oil mass ratio (0:4, 1:3, 1:1, 3:1, and 4:0), and baking temperature (100°C, 110°C, 120°C, 130°C, 140°C, 150°C, 160°C, 170°C, 180°C, 190°C, and 200°C). These variations were used to prepare LPCs in order to analyze their effects on the properties of the final products. For subsequent studies on the effect of baking temperatures (100°C–200°C), the following formulations were selected to simplify the preparation factors: water at 0.25 g/g flour, white granulated sugar at 0.15 g/g flour, and corn oil at 0.4 g/g flour.

### 2.3 Textural Analysis

The textural properties (hardness and fracturability) of the doughs and LPCs were determined using a TA.GEL texture analyzer (Shanghai BosinTech Co. Ltd., China) equipped with a TA/36R probe (cylindrical shape, 36 mm in diameter) for dough and a TA/WEG probe (wedge shape, 30 mm in width, 206 mm in length, 30° cutting angle) for LPCs (Renaldi et al. 2022). The experimental modes were the total test for dough and the single test for LPCs, respectively, for texture profile analysis. The pre‐test speed was 3.00 mm/s, the test speed was 1.00 mm/s, and the post‐test speed was 1.00 mm/s. The compression strains were set at 20% of the original sample height for dough and 3.00 mm for LPCs, respectively. The trigger forces were 0.049 N for dough and 0.098 N for LPCs, respectively. The dwell time was 5.00 s.

### 2.4 Thickness Increase Percentages

The thicknesses of the doughs and the resulting LPCs were measured using a digital vernier caliper (DL90150, Shanghai Deli Stationery Co. Ltd., Shanghai, China). The thickness increase percentage of LPCs was then calculated by dividing the thickness difference between each LPC and its corresponding dough by the dough thickness and multiplying by 100 (Nie et al. 2022).

### 2.5 Weight Loss Percentages

The weights of the doughs and the resulting LPCs were measured using an MTS5000D electronic balance (Meilen, Shenzhen Meifu Electronics Co. Ltd., Shenzhen City, Guangdong Province, China). The weight loss percentage of LPCs was then calculated by dividing the weight difference between each LPC and its corresponding dough by the dough weight and multiplying by 100 (L. Chen et al. 2024).

### 2.6 Water Activity

The water activity values of the LPCs were determined using a handheld water activity instrument (AwTester Basic type, Wisdom Shanghai Instrument Co. Ltd., Shanghai, China) (W. Zhang et al. 2024). Briefly, the LPCs were cut into small particles (size ≤ 2 mm) and then placed into the sample cups, filling to less than two‐thirds of the cup volume. After placing the sample cups into the sample cup base and attaching the sensor probe, the measurement was initiated, and the obtained water activity values were recorded.

### 2.7 Moisture Content

The moisture contents of the LPCs were determined using a drying method (Tao et al. 2022). Briefly, the LPCs were ground in a mortar to a particle size of ≤ 2 mm. Then, the samples were placed in constant‐weight weighing bottles with a sample layer thickness of ≤ 5 mm. The samples were heated in an oven at 103°C ± 1°C until a constant weight was achieved. Finally, the moisture content of the LPCs was calculated using the following equation:

(1) $$\text{Moisture content}\;\left(\%\right)=\frac{m_{1}-m_{2}}{m_{1}-m_{0}}\times 100$$

whereas $m_{0}$ is the weight (g) of the constant‐weight weighing bottle, $m_{1}$ is the total weight (g) of the constant‐weight weighing bottle and the sample before the heating, and $m_{2}$ is the total weight (g) of the constant‐weight weighing bottle and the sample after the heating.

### 2.8 Peroxide Value

The determination of peroxide value (PV) was performed using the potentiometric method (L. Chen et al. 2024; Chu et al. 2026). Briefly, 2.4–3.6 g ($m_{3}$) of the ground LPCs were placed in 250 mL iodine flasks. Then, 30 mL of a trichloromethane (AR, ≥ 99.0%, Sinopharm Chemical Reagent, China) / glacial acetic acid (AR, ≥ 99.5%, Anhui Tiandi High‐Purity Solvent, China) mixture at a volume ratio of 2:3 was added. The flasks were gently shaken, and 1.00 mL of saturated potassium iodide solution (≥ 99.0%, Sinopharm Chemical Reagent) was added, after which the flasks were sealed. After gentle shaking, the flasks were left to stand for 3 min in the dark. Subsequently, 100 mL of water was added and the mixture was shaken. Sodium thiosulfate standard solution ($C_{1}$= 0.002 mol/L, Anpel Experimental Technology Co. Ltd., Shanghai, China) was titrated until the solution color changed to light yellow. Then, 1 mL of starch indicator solution (AR, Sinopharm Chemical Reagent, China) (10 g/L) was added, and the sodium thiosulfate standard solution was continuously titrated until the blue color of the solution disappeared. The total volume ($V_{1}$, mL) of the sodium thiosulfate standard solution used was recorded. The control volume ($V_{0}$, mL) was measured using the same procedure (without sample) and was required to be less than 0.1 mL. The weight loss percentage ($W_{L}$, %) after LPC preparation was obtained as described in Section 2.5. The oil content ($O_{C}$, %) in the dough was calculated as described in Section 2.2. The PV (mmol/kg oil) was calculated using the following equation:

(2) $$\mathrm{PV}\;\left(\text{mmol}/\mathrm{kg}\;\text{oil}\right)=\frac{1000\times C_{1}\times \left(V_{1}-V_{0}\right)}{2\times \frac{m_{3}}{1-W_{L}}\times O_{C}}$$

### 2.9 Acid Value

The acid values of the LPCs were determined using a cold solvent automatic potentiometric titration method (Liu, Mao, and Zhou 2022). Briefly, 2.2–4.4 g ($m_{4}$) of the ground LPCs were placed in 200 mL glass beakers. Then, 70 mL of an ether (AR, ≥ 99.5%, Sinopharm Chemical Reagent) / isopropanol (AR, ≥ 99.7%, General‐Reagent, Shanghai Titan, China) mixture at a volume ratio of 1:1 was added. The mixtures were magnetically stirred until the oil was fully dissolved. Using a ZDJ‐4B automatic potentiometric titrator (INESA Scientific Instrument, China), the samples were titrated with a standard potassium hydroxide solution ($C_{2}$= 0.1 mol/L, Shanghai Anpel, China). The titration volume ($V_{3}$, mL) was recorded. The control volume ($V_{2}$, mL) was measured using the same procedure (without sample). The acid values (mg/g) were calculated according to the following equation:

(3) $$\text{Acid value}=\frac{\left(V_{3}-V_{2}\right)\times C_{2}\times 56.1}{m_{4}}$$

### 2.10 Headspace‐Gas Chromatography–Mass Spectrometry Analysis

The LPCs were cut into small particles (size ≤ 2 mm), and then 2.0 g of the sample was placed into 20 mL headspace bottles. After sealing the bottle with a cap, the small particles were immediately equilibrated at 85°C for 30 min. The volatile organic compounds (VOCs) were analyzed using headspace‐gas chromatography‐ion mobility spectrometry (HS‐GC‐IMS, FlavourSpec Sensitive Analyzer, G.A.S. Gesellschaft für analytische Sensorsysteme mbH, Dortmund, Germany) (J. Chen et al. 2021). The sample injection time was 0.5 min, and the injection syringe temperature was 95°C. The total analysis time was 40.5 min. The chromatographic column was a DB‐WAX column (Restek Stabilwax‐MS, L 30 m, ID 0.32 mm, film thickness 0.25 μm). The carrier and drift gas was N_2_. The IMS temperature was 45°C. The GC unit was held at 40°C for 3 min, then the temperature was raised to 150°C at a rate of 4°C/min, and finally held for 10 min.

The VOCal (Version: 0.2.9 rev) software was used for data analysis with four plugins: Reporter, Gallery Plot, Dynamic Principal Component Analysis (PCA), and Fingerprint Similarity Analysis (FSA). The Reporter plugin was used for difference analysis of the topographic plots. The Gallery Plot plugin was used for fingerprint spectrum comparison and VOC difference analysis. The obtained fingerprint comparison graph was then modified to display the characterized VOCs using Photoshop software. The Dynamic PCA plugin was used for principal component analysis. The FSA plugin was used for fingerprint similarity analysis. A GC × IMS library search was performed to qualitatively analyze VOCs using the built‐in NIST 2014 and IMS databases.

### 2.11 Accelerated Storage Stability Analysis

The LPCs were packed in PA/PE seven‐layer vacuum bags using a FR‐300A heat sealer (Shanghai Yiguang Packaging Equipment Manufacturing Co. Ltd., China) (Shi et al. 2025). The samples were then stored at a relatively high accelerated temperature of 57°C to see their stability at short storage time of 40 days (Lai and Paterson 2020; Nyawo et al. 2025). At designated time points (days 0, 10, 20, 30, and 40), the appearances, colorimetric properties, textural properties, and sensory properties of the samples were analyzed, as described below.

### 2.12 Colorimetric Properties

The colorimetric properties of the preserved LPCs were analyzed using a CR‐400 Chroma Meter colorimeter (Konica Minolta, Tokyo, Japan) following whiteboard calibration (Erdem and Kaya 2021). Lightness (L*) ranged from 0 to 100 (from black to white). Redness‐greenness (a*) ranged from −128 to +128 (from green to red). Yellowness‐blueness (b*) ranged from −128 to +128 (from blue to yellow).

### 2.13 Sensory Properties

Sensory evaluation of the preserved LPCs was conducted using descriptive sensory analysis (Nie et al. 2023). All participants were asked to sign informed consent forms to indicate their agreement to take part in the sensory study and to allow the use of their information. Briefly, 20 panel members (aged 20–35 years, 8 males and 12 females, in good health) were recruited at the hospital to rate the LPCs based on five sensory attributes. For “appearance,” the evaluation criteria were: (i) intact and uniform thickness (20–16 points), (ii) slightly damaged or slightly uneven thickness (15–11 points), and (iii) seriously damaged or uneven thickness (10–0 points). For “color,” the evaluation criteria were: (i) uniform yellow or golden yellow (20–16 points), (ii) slightly uneven color (15–11 points), and (iii) uneven color (10–0 points). For “aroma,” the evaluation criteria were: (i) strong aroma and no rancid smell (20–16 points), (ii) moderate aroma and slight rancid smell (15–11 points), and (iii) almost no aroma and strong rancid smell (10–0 points). For “section structure,” the evaluation criteria were: (i) intact and uniform structure (20–16 points), (ii) slightly damaged or slightly uneven structure (15–11 points), and (iii) seriously damaged or uneven structure (10–0 points). For “taste,” the evaluation criteria were: (i) crispy, good taste, and no graininess (20–16 points), (ii) slightly hard, normal taste, and little graininess (15–11 points), and (iii) hard, bad taste, and heavy graininess (10–0 points). The total score was obtained by summing the scores of the five attributes.

### 2.14 In Vitro Digestion Behaviors

The in vitro digestion behaviors of the LPCs were analyzed using an in vitro digestion model consisting of oral, gastric, and small intestinal phases (Brodkorb et al. 2019; J. M. Xu et al. 2022). Briefly, LPCs prepared at different baking temperatures were chopped into small particles, ground in a mortar, and sieved through a 20‐mesh sieve. Then, 1.0 g ($m_{5}$) of the LPC particles was placed into 50 mL conical flasks. Subsequently, 2.5 mL of preheated (37°C) simulated oral fluid (1.5 mmol/L CaCl_2_ from Shanghai Macklin; 75 U/mL α‐amylase with an activity of 9.4 U/mg solid from Shanghai Yuanye) was added, and the mixture was shaken (100 rpm) at 37°C for 2 min (oral phase). Then, 5 mL of preheated (37°C) simulated gastric fluid (2 g/L NaCl from Shanghai Macklin, 7 mL/L HCl from Sinopharm Chemical Reagent, 3.2 g/L pepsin from porcine gastric mucosa with an activity of 1:3000 from Shanghai Macklin, pH 1.2) was added, and the pH was adjusted to 2.0. The mixture was shaken (100 rpm) at 37°C for 2 h (gastric phase). After adjusting the pH to 7.0, 3.5 mL of porcine bile extract solution (54 mg/mL, Shanghai Macklin, China) and 1.5 mL of salt solution (10 mmol/L CaCl_2_; 150 mmol/L NaCl) were added. After readjusting the pH to 7.0, 2.5 mL of lipase from porcine pancreas (activity: 725 U/mg, Beijing Wokai, China) in phosphate buffer (75 mg/mL, pH 7.0) was added, and the mixture was shaken for 2 h (small intestinal phase).

In the small intestinal phase, the released free fatty acid (FFA) percentage was measured to evaluate the fat digestion behavior of the LPCs. Every 20 min, 0.5 mol/L NaOH ($C_{\text{NaOH}}$, from Shanghai Macklin) was used to adjust the pH back to 7.0. It was assumed that two NaOH molecules were required to neutralize the FFAs released from one triglyceride molecule. The cumulative volume of NaOH ($V_{\text{NaOH}}$, L) was recorded. The blank NaOH volume ($V_{C}$, L) was measured using the same procedure, with the sample replaced by 1 mL of water. The average molecular weight (MW) of triglycerides in corn oil was taken as 871 g/mol (Huda et al. 2024). The released FFA percentage was then calculated according to the following equation:

(4) $$\mathrm{FFA}\;\text{released}\;\left(\%\right)=\frac{\left(V_{\text{NaOH}}-V_{C}\right)\times C_{\text{NaOH}}\times \mathrm{MW}}{\frac{m_{5}}{1-W_{L}}\times O_{C}\times 2}\times 100$$

where 2 indicates that two FFAs were assumed to be released from each triacylglycerol molecule (J. Xu et al. 2024).

The samples in the containers were photographed using a digital camera to observe whether the LPC particles had disappeared (i.e., been digested) or not.

### 2.15 Nutritional Components

The nutritional components of the LPCs were measured by Shanghai WEIPU Testing Technology Group Co. Ltd. (Shanghai, China) and the Instrumental Analysis Center of Shanghai Jiao Tong University (Shanghai, China). Four parallel data sets were obtained from the two organizations (two from each). Briefly, the LPCs were chopped into small particles, ground in a mortar, and sieved through a 20‐mesh sieve. The protein content, fat content, moisture content, ash content, sodium content, energy, and carbohydrate content of the LPC powders were measured and provided by the companies according to procedures accredited by both the China Metrology Accreditation (CMA) and the China National Accreditation Service for Conformity Assessment (CNAS) (Shi et al. 2025).

### 2.16 Statistical Analysis

Three parallel samples were analyzed for each experiment to obtain the data. The data were expressed as mean ± standard deviation (*n* = 3). All data were directly examined using one‐way analysis of variance (ANOVA) followed by Duncan's test in SPSS software (version 26.0, IBM Corp., Armonk, NY, USA) for significance comparison (*p* < 0.05). The assumptions of normality were not verified prior to ANOVA.

## 3 Results and Discussion

### 3.1 Color and Morphological Changes During the LPC Preparation Process

Cookies are generally baked using several raw materials, such as flour, oil, sugar, salt, and water. Therefore, these raw materials were used to prepare LPCs based on a simple baking method consisting of three steps (kneading, molding, and baking), as shown in Figure 1A. The doughs were generally uniform in white color and intact in shape. The obtained LPCs exhibited a relatively uniform yellow color and intact shape. The yellow color was mainly attributed to carbohydrate caramelization and minorly to Maillard reactions during the high‐temperature treatment process (Kim et al. 2021). Moreover, LPCs with intact morphologies on both sides (data not shown) could be prepared even after adjusting the single factors (water addition content, white granulated sugar addition content, corn oil addition content, and fish oil‐to‐corn oil mass ratio) in this work.

The raw material components generally have significant effects on the preparation of cookies. Hardness and fracturability are the most important texture characteristics of cookies (Dhal et al. 2023; Hwang et al. 2016). Among the raw materials (flour, oil, sugar, salt, and water), three component factors (oil, sugar, and water) were selected to analyze their effects on the hardness and fracturability of the doughs and LPCs. Corn oil is a popular vegetable oil worldwide (Boukid and Rosentrater 2024), while fish oil is rich in omega‐3 polyunsaturated fatty acids and offers various health benefits (J. Xu et al. 2024). Therefore, the effects of four component addition parameters (water, sugar, corn oil, and fish oil/corn oil mass ratio) on the textural properties (Figure 1B,I) of the doughs and LPCs were analyzed in the subsequent studies prior to further research on the effect of baking temperature.

### 3.2 Effect of Water Addition Content on the Textural Properties of Doughs and LPCs

The effect of water addition content (0.15, 0.25, 0.35, 0.45, and 0.55 g/g flour) on the preparation and textural properties of doughs and LPCs was analyzed. At water addition levels below 0.15 or above 0.55 g/g flour, the mixture of sugar/salt solution, corn oil, and low‐protein flour could not form good doughs (data not shown). The hardness (Figure 1B) and fracturability (Figure 1C) of the doughs decreased logarithmically with increasing water content (0.15–0.55 g/g flour). This was because the doughs became softer after adding more water. However, the hardness (Figure 1B) and fracturability (Figure 1C) of the LPCs increased logarithmically and then decreased with increasing water content. The percentage of corn oil decreased as the water addition increased during the preparation process. Previous work suggested that the presence of fat could induce air incorporation during dough making (Pareyt et al. 2009). Therefore, the decreasing oil content resulted in less incorporated air in the doughs and fewer air cells in the LPCs (Pareyt et al. 2009), which in turn increased the hardness and fracturability of the LPCs.

### 3.3 Effect of Sugar Addition Content on the Textural Properties of Doughs and LPCs

The effect of white granulated sugar addition content (0.00, 0.05, 0.15, 0.25, 0.35, and 0.45 g/g flour) on the preparation and textural properties of doughs and LPCs was analyzed. At sugar addition levels above 0.45 g/g flour, the mixture of sugar/salt solution, corn oil, and low‐protein flour could not form good doughs (data not shown). The hardness (Figure 1D) and fracturability (Figure 1E) of both the doughs and LPCs increased and then decreased with increasing sugar content (0.00–0.45 g/g flour). Previous work suggested that increasing sugar levels could increase the porosity of cookies (Pareyt et al. 2009). White granulated sugar exists in a viscous molecular form in doughs and in a crystalline form in cookies. Our results suggested that an appropriate amount of sugar (≤ 0.35 g/g flour) in the doughs (in viscous molecular form) and LPCs (in crystalline form) could strengthen their structures, thereby increasing their hardness and fracturability. However, excessive sugar (≥ 0.45 g/g flour) could disrupt the structures of the doughs (even inhibiting dough formation) and LPCs, consequently decreasing their hardness and fracturability.

### 3.4 Effect of Corn Oil Addition Content on the Textural Properties of Doughs and LPCs

The effect of corn oil addition content (0.2, 0.3, 0.4, 0.5, 0.6, and 0.7 g/g flour) on the preparation and textural properties of doughs and LPCs was analyzed. At corn oil addition levels below 0.2 or above 0.7 g/g flour, the mixture of sugar/salt solution, corn oil, and low‐protein flour could not form good doughs (data not shown). The hardness (Figure 1F) and fracturability (Figure 1G) of both the doughs and LPCs decreased linearly and then showed no obvious changes with increasing corn oil content (0.2–0.7 g/g flour). Increasing oil addition (below 0.6 g/g flour) induced more incorporated air in the doughs and more air cells in the LPCs (Pareyt et al. 2009), thereby decreasing the hardness and fracturability of the LPCs. However, excessive oil (≥ 0.6 g/g flour) could not further induce more incorporated air in the doughs or more air cells in the LPCs, and therefore showed no obvious effect on the hardness and fracturability of the LPCs.

### 3.5 Effect of Fish Oil/Corn Oil Mass Ratio on the Textural Properties of Doughs and LPCs

Oil blend is an important consideration to improve the properties of cookies (Hitlamani et al. 2025). The effect of fish oil‐to‐corn oil mass ratio (0:4, 1:3, 1:1, 3:1, and 4:0) on the preparation and textural properties of doughs and LPCs was analyzed. At fish oil/corn oil mass ratios ranging from 0:4 to 4:0, both doughs and LPCs could be successfully prepared (data not shown). The hardness (Figure 1H) and fracturability (Figure 1I) of the doughs and LPCs increased and then decreased with increasing fish oil‐to‐corn oil mass ratio. Notably, the ratio of 1:1 resulted in the highest hardness values (146.0 N and 44.7 N) and fracturability values (146.0 N and 44.0 N) for both the doughs and LPCs. This suggested that fish oil and corn oil synergistically enhanced the textural properties (hardness and fracturability) of both the doughs and LPCs. Therefore, further work is needed to elucidate the underlying synergistic mechanism.

### 3.6 Effect of Baking Temperature on the Color and Morphologies of LPCs

**FIGURE 2:**
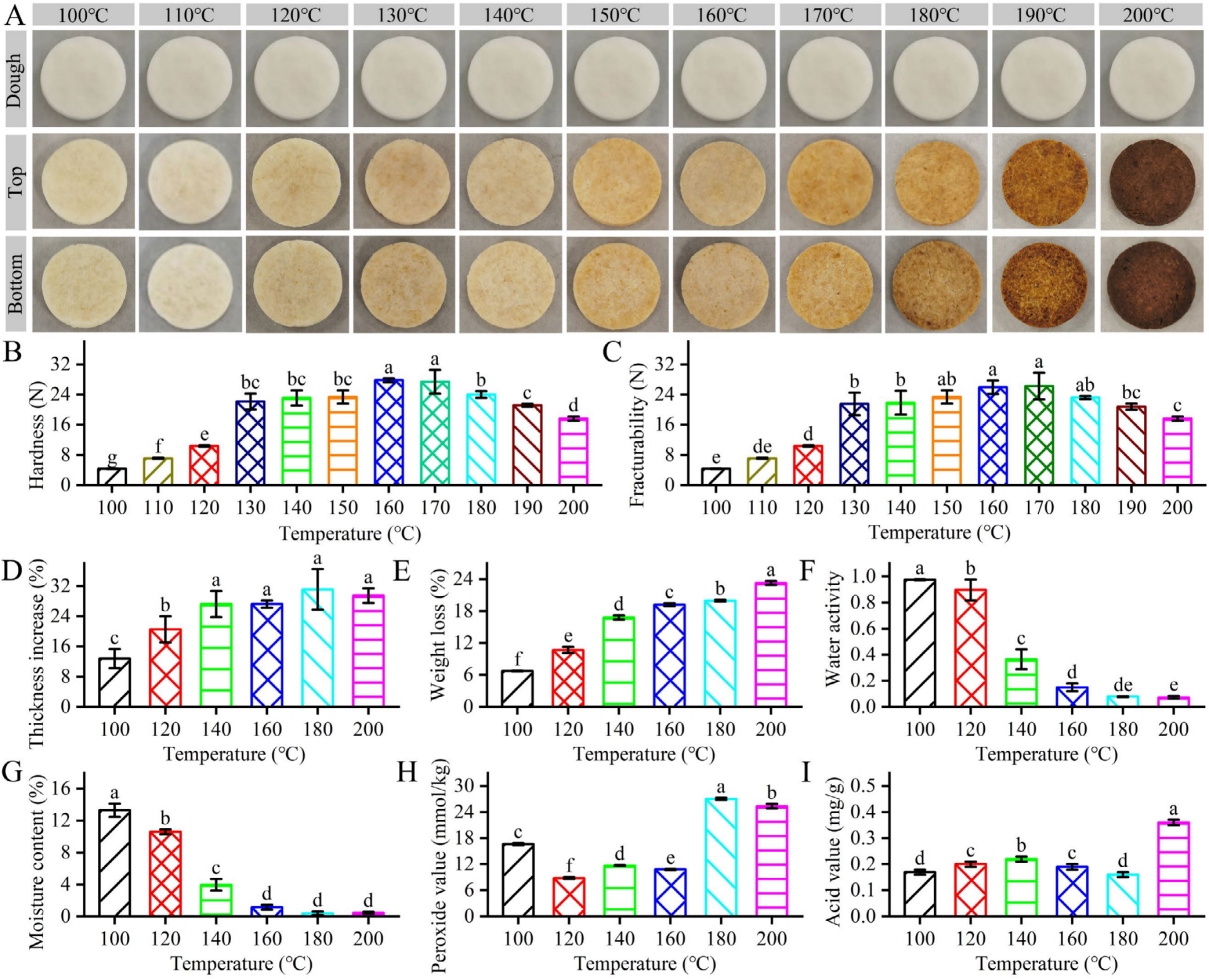
Effects of baking temperatures on the physicochemical properties of the LPCs. (A) Appearances of the doughs and SPLCs (top and bottom sides). (B) Hardness. (C) Fracturability. (D) Thickness increase percentages. (E) Weight loss. (F) Water activity. (G) Moisture contents. (H) Peroxide values (mmol/kg oil). (I) Acid values. The temperature intervals for (A–C) and (D–I) were 10°C and 20°C, respectively. The columns and error bars indicate the average values and standard deviation (*n* = 3). Different letters on the columns in each image indicate significant differences (*p* < 0.05) using one‐way ANOVA and Duncan's test.

Different baking temperatures (100°C–200°C) were used to prepare the LPCs. As shown in Figure 2A, all doughs were white, while the obtained LPCs exhibited colors ranging from light yellow to brown with increasing baking temperatures. Moreover, both the top and bottom sides of the LPCs showed similar colors, which was attributed to the flipping‐over step during the baking process. High‐temperature treatment induced mainly carbohydrate caramelization and minorly Maillard reactions due to low protein contents in the flours, which contributed to the formation of cookie color (Kim et al. 2021). This explained why higher baking temperatures resulted in darker cookie colors (Figure 2A). In addition, all the obtained LPCs displayed intact morphologies on both sides.

### 3.7 Effect of Baking Temperature on the Textural and Water‐Related Properties of LPCs

With increasing baking temperature, the hardness (Figure 2B: 4.4–27.8 N) and fracturability (Figure 2C: 4.4–26.2 N) increased from 100°C to 160°C, remained unchanged from 160°C to 170°C, and then decreased from 170°C to 200°C. As shown in Figure 2D, the thickness increase percentages of the LPCs (relative to the doughs) increased with baking temperature from 100°C to 140°C and then remained unchanged from 140°C to 200°C. With rising baking temperature, the weight loss percentages (Figure 2E) increased logarithmically, while water activity (Figure 2F) and moisture content (Figure 2G) decreased exponentially.

Moisture in the dough turns into steam at approximately 100°C, causing the dough to rise and form cookies (Dhal et al. 2023). Starch gelatinization contributes to the formation of the food matrix (Liu, Yang, et al. 2022). Moreover, higher baking temperatures induce faster starch gelatinization in cookies (Dhal et al. 2023), which enhances the textural properties (e.g., hardness and fracturability) of the resulting cookies. Both moisture steaming and starch gelatinization affect the properties of the final cookies. It is reasonable that higher temperatures lead to greater water evaporation during baking, resulting in drier cookies and positively influencing hardness and fracturability. The observed increase in thickness (Figure 2D) was attributed to the enlargement of cell sizes within the cookies (Pareyt et al. 2009), which might negatively affect hardness and fracturability. Therefore, decreasing moisture content (Figure 2G) and starch gelatinization were the main factors responsible for the increases in hardness (Figure 2B) and fracturability (Figure 2C). Starch gelatinization occurred at baking temperatures of ≥ 130°C, which significantly increased the hardness (Figure 2B) and fracturability (Figure 2C) of the obtained LPCs. In addition, the decreasing moisture content led to increased thickness (Figure 2D), which was the primary reason for the reduction in hardness (Figure 2B) and fracturability (Figure 2C) at baking temperatures of 170°C–200°C.

The increase in cookie thickness (Figure 2D) during baking involved the generation of gases from chemical leaveners and water vaporization, which together form a porous structure within the cookies (HadiNezhad and Butler 2009). As the baking temperature increased from 100°C to 140°C, gas production increased, leading to greater cookie thickness. At 140°C, all chemical leaveners and water were sufficiently heated to generate the maximum amount of gas. Consequently, cookie thickness remained unchanged across the temperature range of 140°C–200°C.

### 3.8 Effect of Baking Temperature on the Peroxide and Acid Values of LPCs

To evaluate the quality of corn oil in LPCs prepared at different baking temperatures, the PVs (Figure 2H) and acid values (Figure 2I) were determined. The PVs depended on baking temperature as follows: 180°C (27.0 ± 0.2 mmol/kg oil) > 200°C (25.3 ± 0.5 mmol/kg oil) > 100°C (16.6 ± 0.2 mmol/kg oil) > 140°C (11.7 ± 0.1 mmol/kg oil) > 160°C (10.8 ± 0.1 mmol/kg oil) > 120°C (8.8 ± 0.2 mmol/kg oil). The acid values also varied with baking temperature: 200°C (0.36 ± 0.01 mg/g) > 140°C (0.22 ± 0.01 mg/g) > 120°C (0.20 ± 0.01 mg/g) > 160°C (0.19 ± 0.01 mg/g) > 100°C (0.17 ± 0.01 mg/g) > 180°C (0.16 ± 0.01 mg/g). Food products with PVs of < 10, 10–20, and > 20 mmol/kg oil are classified as having low, moderate, and high oxidation states, respectively (Sayed Ahmad et al. 2018). The acid values obtained were similar to those (0.15–0.38 mg/g) reported for cookies containing essential oils (Hematian Sourki et al. 2021). Previous work suggested that both PVs and acid values of school meal biscuits increased with increasing baking temperatures in the range of 180°C–200°C (Abd El‐Baset and Almoselhy 2023). Higher temperatures caused increased lipid peroxidation (Shahidi and Hossain 2022), leading to elevated PVs and acid values. According to these results (Figure 2H,I), moderate baking temperatures (120°C–160°C) were necessary to produce LPCs with good quality (PVs: 8.8–11.7 mmol/kg oil; acid values: 0.19–0.22 mg/g). The effects of baking temperature on the PVs and acid values of LPCs differed from those reported for school meal biscuits (Abd El‐Baset and Almoselhy 2023). This discrepancy might be attributed to the different food matrix (low‐protein) compared with regular biscuits.

### 3.9 Effect of Baking Temperature on the VOCs of LPCs

HS‐GC‐IMS was applied to analyze the VOCs in LPCs prepared at different baking temperatures. The VOCs were identified using two‐dimensional separations (drift time and retention time) (J. Chen et al. 2021). Equal masses of LPCs were placed into headspace bottles for HS‐GC‐IMS analysis, allowing comparison of VOCs across different baking temperatures. As shown in the representative topographic plots (Figure S1A) and topographic subtraction plots (Figure S1B), 126 areas (spots) with distinct drift times and retention times were identified in the LPCs. Based on the NIST and IMS databases, 74 VOCs were identified in the topographic plots (indicated by arrows 1–74 in Figure S1A) and were listed in Table S1. Previous work suggested that the key VOCs in bakery foods (e.g., biscuits, bread, crackers, and cakes) include pyrazines, pyrroles, ketones, lactones, furans, aldehydes, alcohols, esters, and acids (Sonmezdag et al. 2022). The 74 VOCs identified in this study (Table S1) comprised 30 aldehydes, 14 ketones, 14 alcohols, 8 esters, 4 acids, 3 furans, and 1 benzene. These VOC types were consistent with those previously detected in bakery foods (Sonmezdag et al. 2022).

**FIGURE 3:**
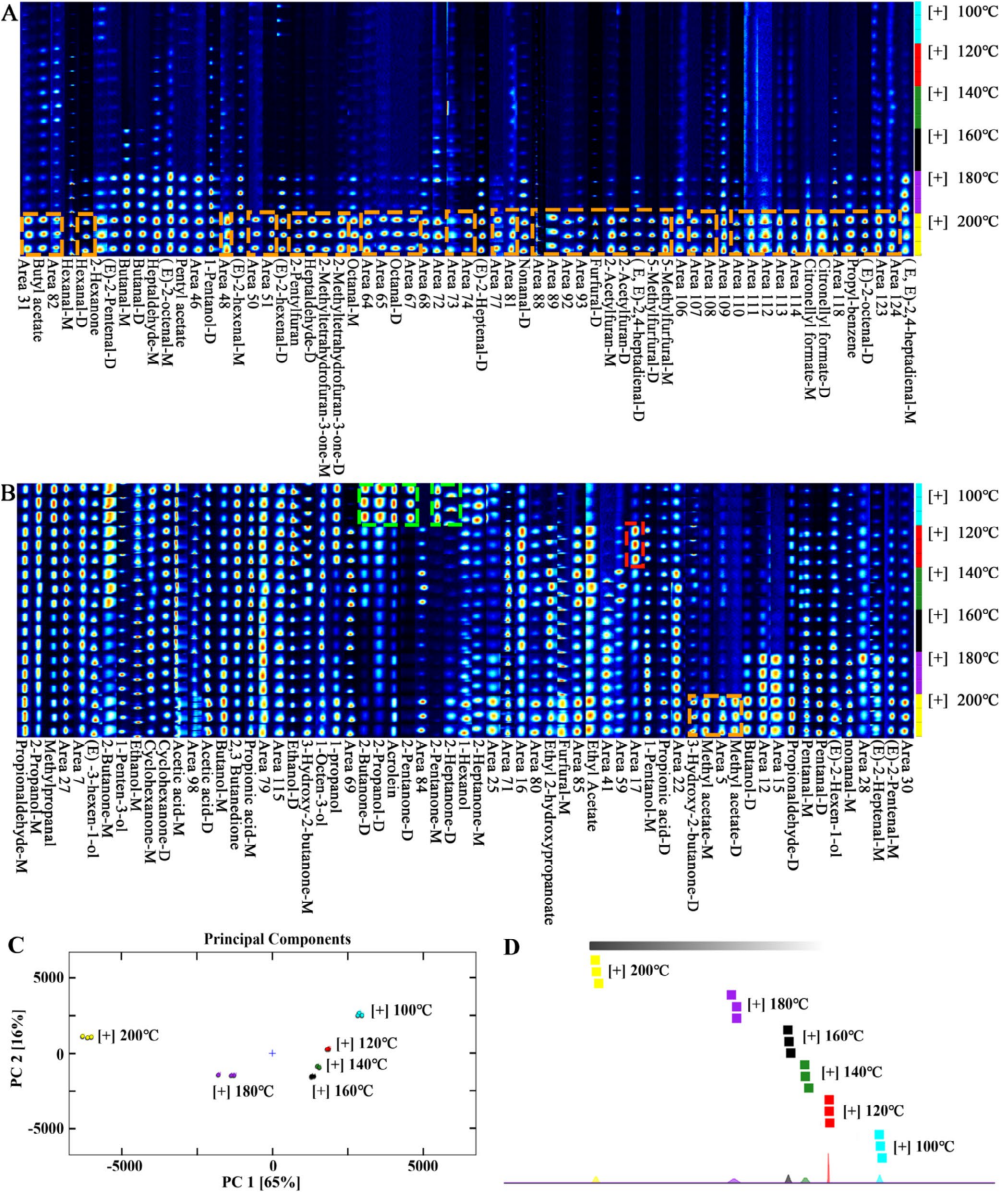
VOC analysis of the LPCs with different baking temperatures using HS‐GC‐IMS. (A, B): VOCs fingerprint comparisons. The redder the area is, the larger the quantity of VOC is. Three parallel samples were analyzed for each baking temperature (*n* = 3). Each row and column represent all the signals for the same sample and VOC, respectively. The orange dashed, green dashed, and red dotted squares represent the characterized VOCs for the LPCs with the baking temperatures of 200°C, 100°C, and 120°C, respectively. (C): Principal component analysis. (D): Fingerprint similarity analysis.

High temperatures promoted the increase of ketones, aldehydes, and acids in milk powders (Feng et al. 2021). To analyze the effects of high temperatures on VOCs, fingerprint profile comparisons of the 126 identified areas (spots) were performed using the Gallery Plot plugin of the HS‐GC‐IMS commercial VOCal software. As shown in Figure 3A,B, many VOCs appeared only at high baking temperatures (≥ 180°C), such as (E, E)‐2, 4‐heptadienal‐M (Figure 3A). Some VOCs were present at all baking temperatures, such as propionaldehyde‐M (Figure 3B). Two VOCs (2‐pentanone‐D and 2‐heptanone‐D in Figure 3B) disappeared at moderate and high baking temperatures (≥ 120°C). Conversely, some VOCs appeared at moderate and high baking temperatures (≥ 120°C), such as (E)‐2‐heptenal‐M and (E)‐2‐pentenal‐M (Figure 3B).

For LPCs baked at 100°C, six characteristic VOCs were identified (2‐butanone‐D; 2‐propanol‐D; acrolein; 2‐pentanone‐D; 2‐pentanone‐M; and 2‐heptanone‐D) (Figure 3B). Acrolein is a key volatile responsible for flavor deterioration during the early stages of fish oil oxidation (Shibata et al. 2018). For LPCs baked at 120°C, one characteristic VOC (Area 17) was identified (Figure 3B). For LPCs baked at 200°C, 47 characteristic VOCs were identified (indicated by orange dashed squares in Figure 3A), which were associated with the dark color of the LPCs (Figure 2A). Among these 47 VOCs, 26 were identified, including 3‐hydroxy‐2‐butanone‐D; methyl acetate‐M; methyl acetate‐D; hexanal‐D; butyl acetate; 2‐pentylfuran; heptaldehyde‐D; 2‐methyltetrahydrofuran‐3‐one‐M; 2‐methyltetrahydrofuran‐3‐one‐D; octanal‐D; furfural‐D; 2‐acetylfuran‐M; 2‐acetylfuran‐D; (E, E)‐2, 4‐heptadienal‐D; 5‐methylfurfural‐D; 5‐methylfurfural‐M; citronellyl formate‐M; citronellyl formate‐D; propyl‐benzene; and (E)‐2‐octenal‐D.

Furan and its derivatives are prevalent contaminants in thermally processed foods (Meng et al. 2024). Therefore, compounds such as 2‐pentylfuran, 2‐methyltetrahydrofuran‐3‐one‐M, 2‐methyltetrahydrofuran‐3‐one‐D, furfural‐D, 2‐acetylfuran‐M, 2‐acetylfuran‐D, (E, E)‐2, 4‐heptadienal‐D, 5‐methylfurfural‐D, and 5‐methylfurfural‐M may pose potential safety concerns for LPCs baked at 200°C. According to the European Food Safety Authority (EFSA), the median dietary intake of furan for adults aged 18–65 years ranges from 320 to 350 ng/kg body weight per day (Chain et al. 2017). For an adult with a body weight of 50 kg, this corresponded to a median intake of 16.0–17.5 μg/day. Therefore, further characterization of furan in LPCs is necessary to assess the safety of LPCs baked at 200°C.

Considering that moderate baking temperatures (120°C–160°C) are necessary to produce LPCs with good corn oil quality, based on PVs (Figure 2H) and acid values (Figure 2I), the VOCs that appeared at these moderate temperatures might be the key contributors to the desirable flavor of the LPCs.

The VOC differences in LPCs prepared at different baking temperatures were analyzed using PCA (Figure 3B) and FSA (Figure 3C) based on the area signal intensities. PCA is a multivariate data analysis method designed for multidimensional datasets with quantitative variables (Szymańska‐Chargot et al. 2024). FSA is used to analyze the similarity of VOC fingerprints based on Euclidean distance (Li et al. 2023). Both PCA (Figure 3B) and FSA (Figure 3C) showed that the LPCs were independent and distinguishable from one another. Therefore, baking temperatures induced different effects on the VOC intensities of the LPCs.

### 3.10 Effects of Baking Temperature on the Accelerated Stability of LPCs

**FIGURE 4:**
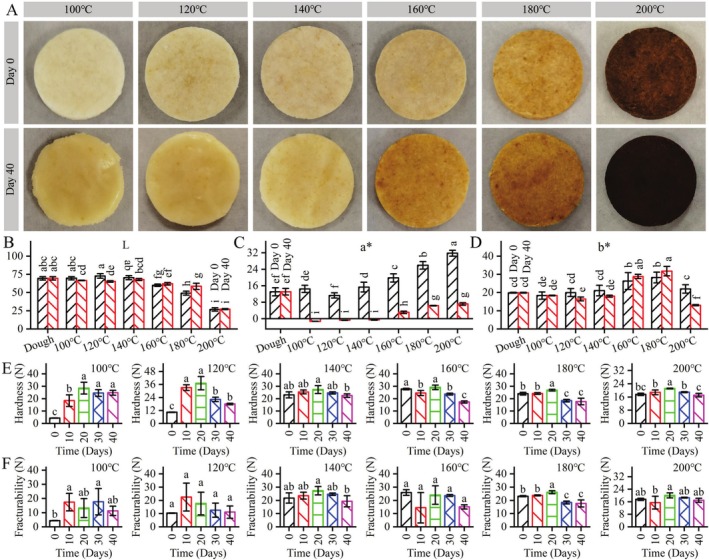
Appearances, colorimetry, and textural analyses of the LPCs with different baking temperatures at the accelerated storage condition. The LPCs were sealed and then stored at 57°C for 40 days. (A) Appearances using a digital camera. (B) Color parameter lightness L*. (C) Color parameter chroma a*. (D) Color parameter chroma b*. (E) Hardness. (F) Fracturability. The columns and error bars indicate the average values and standard deviation (*n* = 3). Different letters on the columns in each image indicate significant differences (*p* < 0.05) using one‐way ANOVA and Duncan's test.

The oxidative stability of corn oil in LPCs prepared at different baking temperatures was analyzed by storing the sealed LPCs at 57°C for 40 days (Lai and Paterson 2020). As shown in Figure 4A, the colors of the LPCs became darker over time. Colorimetric analysis (Figure 4B,D) revealed that lightness (L*, Figure 4B), redness‐greenness (a*, Figure 4C), and yellowness‐blueness (b*, Figure 4D) were dependent on the baking temperature. After storage, a significant decrease in redness‐greenness was observed for all LPCs, regardless of baking temperature (100°C–200°C).

The hardness (Figure 4E) and fracturability (Figure 4F) were measured to evaluate the textural stability of the LPCs. The textural stability depended on both baking temperature and storage duration. For LPCs baked at 100°C–120°C, hardness and fracturability increased after 10 days of storage. For LPCs baked at 140°C, 180°C, and 200°C, hardness and fracturability increased during the first 20 days of storage and then decreased between 20 and 40 days. For LPCs baked at 160°C, hardness and fracturability decreased from 0 to 10 days, increased from 10 to 20 days, and then decreased again from 20 to 40 days of storage.

### 3.11 Effects of Baking Temperature on the Sensory Stability of LPCs

**FIGURE 5:**
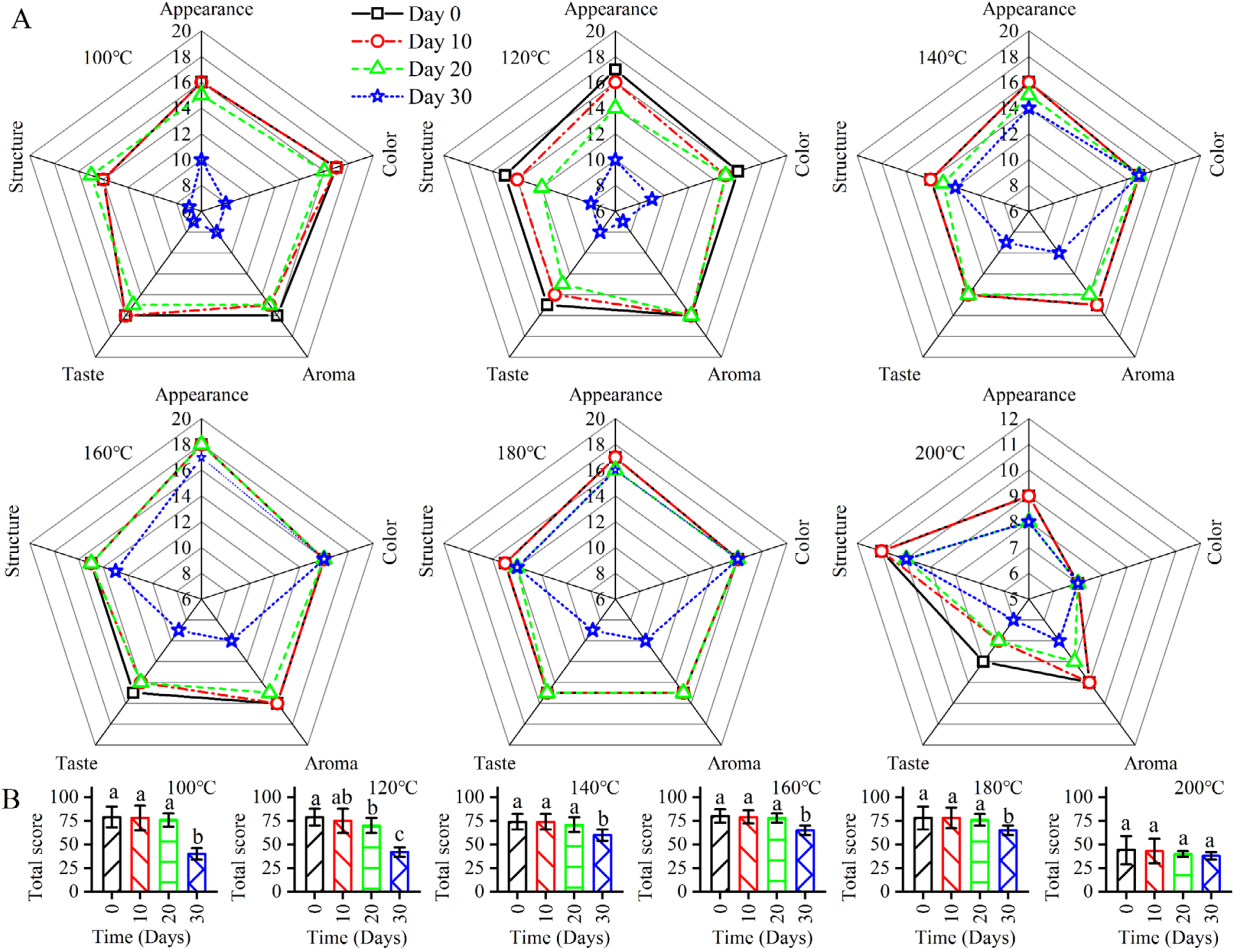
Sensory evaluation of the LPCs with different baking temperatures at accelerated storage conditions. The LPCs were sealed and then stored at 57°C for 30 days. No sensory evaluation data were summarized for day 40 because the LPCs had an obvious rancid smell. (A) Radar graphs. (B) Total scores. Data (mean ± standard deviation) were obtained from the evaluation scores from 20 panel members. Different letters on the columns in each image indicate significant differences (*p* < 0.05) using one‐way ANOVA and Duncan's test.

The effects of baking temperature and storage duration on the sensory properties of the LPCs were evaluated, as shown in Figure 5. All sensory attributes (appearance, color, aroma, taste, and section structure) were dependent on both baking temperature and storage duration (Figure 5A). The freshly prepared LPC baked at 200°C exhibited undesirable sensory performance in four attributes (appearance, color, aroma, and taste). By day 40, all LPCs had developed obvious rancid odors. For LPCs baked at 100°C–120°C, all five sensory attributes significantly decreased by day 30. For LPCs baked at 140°C–180°C, only two sensory attributes (taste and aroma) significantly decreased by day 30. As shown in Figure 5B, the total sensory scores of all LPCs showed no obvious changes from day 0 to day 20, but then significantly decreased by day 30. These results suggest that baking temperatures of 140°C–180°C conferred the best sensory stability to the LPCs compared to other temperatures. The LPC baked at 200°C showed significantly lower sensory scores than the others, which may be mainly attributed to the production of the 47 characteristic VOCs (indicated by orange dashed squares in Figure 3A).

### 3.12 Effect of Baking Temperature on the In Vitro Digestion Behaviors of LPCs

**FIGURE 6:**
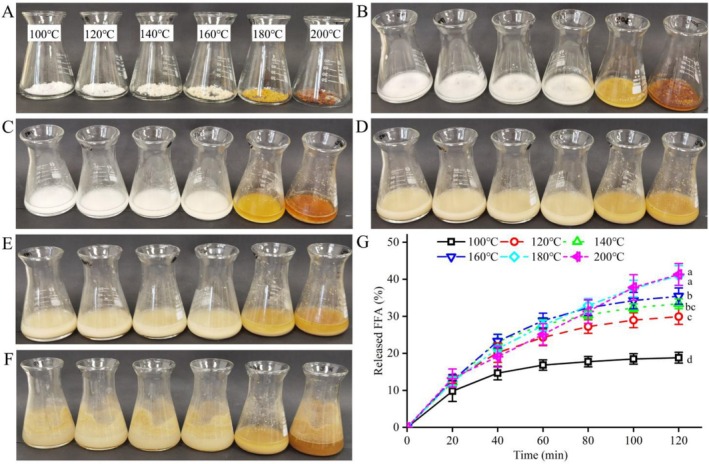
In vitro behaviors of the LPCs with different baking temperatures in the simulated gastrointestinal model at room temperature. (A–F): Digital camera images of the LPCs in the simulated digestion behaviors. (A): The LPCs before the oral phase. (B): The samples after adding the simulated oral fluid. (C): The samples after adding the simulated gastric fluid. (D): The samples after adding the porcine bile extract solution. (E): The samples after adding the salt solution. (F): The samples after the small intestinal phase. (G): Free fatty acid (FFA) release percentages of the LPCs in the small intestinal phase. Data (mean ± standard deviation) were obtained from three parallel samples for each baking temperature. Different letters indicate significant differences (*p* < 0.05) for the accumulative FFA release percentages at 120 min.

The digestion behaviors of LPCs prepared at different baking temperatures were analyzed using an in vitro digestion model (Brodkorb et al. 2019; J. M. Xu et al. 2022). As shown in Figure 6A,F, LPC particles remained present even after the entire digestion process. Therefore, the LPC particles were not fully digested in the in vitro digestion system.

The released FFA percentages of the LPCs in the small intestinal phase were determined, as shown in Figure 6G. These percentages were dependent on baking temperature. The accumulated FFA released percentages after 2 h in the small intestinal phase increased with rising baking temperature: 100°C (18.8% ± 1.5%) < 120°C (29.9% ± 2.1%) ≤ 140°C (33.4% ± 1.3%) ≤ 160°C (35.4% ± 2.2%) < 180°C (41.0% ± 2.6%)≈200°C (41.3% ± 3.0%). These FFA released percentages were comparable to or lower than those (25.7%–64.8%) reported for fish oil‐loaded gelatin‐polysaccharide coacervated powders (Peng et al. 2024). However, those coacervated powders were fully dissolved in gastric fluid (Peng et al. 2024). A possible reason for this difference is that the LPC particles were difficult to dissolve in the digestion fluids, which may be mainly attributed to the high lipid entrapment ability of the food matrix (Shahidi and Pan 2022). The ingredients of the low‐protein steamed bun flour are complex, including wheat starch, potato starch, corn starch, acetate starch, pea starch, edible glucose, gluconic acid‐δ‐lactone, calcium carbonate, and sodium bicarbonate. Therefore, further studies are needed to analyze the underlying matrix effects on the in vitro digestion behaviors of LPCs.

### 3.13 Nutritional Component Analysis of LPCs

Based on the PVs (Figure 2H) and acid values (Figure 2I) results, moderate baking temperatures (120°C–160°C) were necessary to produce LPCs with good corn oil quality. The sensory stability results (Figure 5) indicated that baking temperatures of 140°C–180°C conferred the best sensory stability to the LPCs compared to other temperatures. In addition, the LPC baked at 160°C exhibited lower water activity (Figure 2F) and moisture content (Figure 2G) than that baked at 140°C. Generally, the lower the water activity and moisture content of a bakery food, the longer its shelf life. Finally, the LPC baked at 160°C showed a slightly higher accumulative FFA release percentage (Figure 6G) than that baked at 140°C. Therefore, 160°C was selected as the representative baking temperature for the preparation of LPCs in this work.

The nutritional components of the LPC baked at 160°C were determined by third‐party testing companies. The moisture content, ash content, protein content, carbohydrate content, fat content, and sodium content were 1.89 ± 0.12 g/100 g, 0.62 ± 0.06 g/100 g, 0.27 ± 0.01 g/100 g, 72.03 ± 0.36 g/100 g, 25.00 ± 0.36 g/100 g, and 182 ± 15 mg/100 g, respectively. The moisture and ash contents were lower than those of normal cookies (7.7–13.0 g/100 g for moisture; 1.3–2.2 g/100 g for ash) reported in a previous work (Din et al. 2024). The protein content of the cookies was generally dependent on the raw material composition (low‐protein flour: corn oil:sugar:salt = 1:0.4:0.15:0.005) and the moisture content (1.89 g/100 g was close to the measured result in our laboratory) of the final product (Figure 2G). Considering the low‐protein flour has a protein content of 0.47 g/100 g, the LPC baked at 160°C was expected to have a protein content of 0.30 g/100 g, which was close to the measured value (0.27 g/100 g) by third‐party testing companies.

Given that samples baked at 100°C–140°C had higher moisture contents than those baked at 160°C, their protein contents were expected to be even lower (< 0.27 g/100 g). In contrast, LPCs baked at 180°C–200°C showed moisture contents comparable to the 160°C sample, suggesting similar protein levels (~0.27 g/100 g). Based on these observations, it is reasonable to assume that all LPCs prepared within the baking temperature range of 100°C–200°C maintain a low protein content.

The energy content was 2150 ± 14.3 KJ/100 g (514 ± 3.4 Kcal/100 g), which was comparable to that of wheat‐red kidney bean biscuits (1595–1628 KJ/100 g) (Roy et al. 2020) and shortcake biscuits (2223 ± 13.5 KJ/100 g) (Zielińska and Pankiewicz 2020) reported in previous studies. Previous research indicated that commercial LPCs had protein contents of 0.4 (0.1–0.8) g/100 g, fat contents of 19.1 (1.5–49.4) g/100 g, carbohydrate contents of 75.1 (48.3–87.7) g/100 g, and energy values of 478 (395–639) Kcal/100 g (Pena et al. 2015). Therefore, the LPCs obtained in this work are excellent LPCs with high energy content.

Protein, particularly gluten, plays a crucial role in determining the structural integrity and texture of baked products (Ortolan and Steel 2017). The low‐protein steamed bun flour mainly consists of wheat starch, potato starch, corn starch, acetylated starch, pea starch, edible glucose, glucono‐delta‐lactone, calcium carbonate, and sodium bicarbonate (Zi et al. 2026). Given that the obtained LPCs had a low protein content of 0.27 g/100 g, the food additives in the low‐protein steamed bun flour may play a crucial role in determining the structural integrity and texture of the baked LPCs. Glucono‐delta‐lactone is a food additive used in cheese manufacturing to create a softer cheese texture by adjusting pH, enhancing milk coagulation, facilitating casein breakdown, and promoting curd formation (Al‐Hatim et al. 2021). Notably, it can also be used as a leavening agent in bakery products to improve texture (Mohd Shukri and Cheng 2023). Calcium carbonate and sodium bicarbonate are also leavening agents that improve the texture of bakery products (Kukurová and Ciesarová 2024). Therefore, the application of these three food additives compensated for the issues caused by the low protein and gluten contents in the LPCs.

## 4 Conclusions

In this work, low‐protein cookies (LPCs) with low protein content and high energy were successfully developed. This work provides useful information for understanding the effects of composition and baking temperature on the properties of low‐protein foods. It also offers effective low‐protein food options for patients with inherited amino acid metabolism disorders, inherited organic acid metabolism disorders, inherited urea cycle disorders, and chronic kidney disease, allowing them to enjoy the pleasure of cookies as part of a normal diet. The LPC baked at 160°C had a carbohydrate content of 72.03 g/100 g, a fat content of 25.00 g/100 g, and other nutrients. Therefore, these LPCs can fulfill the nutritional requirements for carbohydrates, fats, and other nutrients in such patients. Patients could consume these LPCs along with other protein‐based foods (e.g., phenylalanine‐free foods for special medical purposes) to meet all their nutritional needs (e.g., for patients with phenylketonuria).

Due to the limitations of this work, further research is needed to improve upon these findings in the future. First, this work oversimplified the complex nature of cookie formulation and neglected potential interactions between critical variables, such as water content × lipid concentration, sugar level × baking temperature, and oil composition × oxidation kinetics. Further studies on the effects of molecular interactions and related parameters should be conducted for the development of LPCs. Second, the key VOCs for LPCs should be further analyzed and characterized using external standards or gas chromatography–mass spectrometry. Third, the in vitro digestion behavior of LPC particles should be further evaluated by analyzing particle size distribution and enzymatic accessibility. Fourth, in vivo digestion studies of LPCs would be useful for understanding the relationships between preparation parameters and nutritional behaviors. Fifth, the underlying matrix effects on the in vitro digestion behaviors of LPCs should be explored to guide the development of low‐protein foods. Sixth, scale‐up processing of LPCs should be investigated to develop commercial products for the dietary management of chronic kidney diseases and inherited metabolic disorders. Finally, it would be interesting to explore how reduced protein content influences the structural, nutritional, and functional properties of the cookies.

## Author Contributions

**Ye Zi:** investigation. **Wei Cai:** supervision. **Zhenfeng Liu:** investigation. **Jian Zhong:** conceptualization, data curation, supervision, funding acquisition, writing – review and editing. **Cuiping Shi:** data curation, investigation, writing – original draft, methodology.

## Funding

This research has been supported by a research grant from the Shanghai Municipal 3 Year Action Plan for Strengthening the Construction of Public Health System (2023–2025) Discipline Leader Project (GWVI‐11.2‐XD19).

## Conflicts of Interest

The authors declare no conflicts of interest.

## Supporting information


**Table S1:** Comparisons of the detected volatile organic compounds (VOCs) in the special low protein cookies (SLPC, *n* = 3) with different baking temperatures by headspace‐gas chromatography–mass spectrometry (HS‐GC‐IMS).
**Figure S1:** Volatile fingerprints of the LPCs with different baking temperatures using headspace‐gas chromatography‐ion mobility spectrometry (HS‐GC‐IMS). (A): Topographic plots. Numbers (1–73) indicate the areas (spots, peaks) that are the identified volatile organic compounds (VOCs) characterized by the retention time (on the y‐axis), the relative drift time (on the x‐axis), and the intensity of the signals. The redder the area is, the larger the quantity of VOC is. The information on the number 74 was written in the top‐right corner. (B): Topographic subtraction plots. They were obtained by subtracting the SLPC plot with 100°C (The leftmost image) from the other sample plots. In the topographic subtraction plots (The 5 images on the right), the red and blue colors indicate more or less, respectively, contents than the SLPC plot (The leftmost image) with 100°C.

## Data Availability

The data that support the findings of this study are available from the corresponding author upon reasonable request.
