# Supplementary material for: Effects of Composition and Baking Temperature on the Properties of Low‐Protein Cookies
Source: Food Sci Nutr. 2026 Jun 30;14(7):e72069. doi: 10.1002/fsn3.72069 (PMC13316454; doi:10.1002/fsn3.72069)
Supplement: Supplementary file 1 — Table S1: Comparisons of the detected volatile organic compounds (VOCs) in the special low protein cookies (SLPC, n = 3) with different baking temperatures by headspace‐gas chromatography–mass spectrometry (HS‐GC‐IMS). Figure S1: Volatile fingerprints of the LPCs with different baking temperatures using headspace‐gas chromatography‐ion mobility spectrometry (HS‐GC‐IMS). (A): Topographic plots. Numbers (1–73) indicate the areas (spots, peaks) that are the identified volatile organic compounds (VOCs) characterized by the retention time (on the y‐axis), the relative drift time (on the x‐axis), and the intensity of the signals. The redder the area is, the larger the quantity of VOC is. The information on the number 74 was written in the top‐right corner. (B): Topographic subtraction plots. They were obtained by subtracting the SLPC plot with 100°C (The leftmost image) from the other sample plots. In the topographic subtraction plots (The 5 images on the right), the red and blue colors indicate more or less, respectively, contents than the SLPC plot (The leftmost image) with 100°C. [file FSN3-14-e72069-s001.docx]

**Supplementary Materials**

**Effects of composition and baking temperature on the properties of low-protein cookies**

Cuiping Shi^1,2,3^, Ye Zi^1,2,3^, Zhenfeng Liu^4^, Wei Cai^1,2,3,*^, Jian Zhong^1,2,3,*^

^1^ Shanghai Key Laboratory of Pediatric Gastroenterology and Nutrition, Xinhua Hospital, Shanghai Jiao Tong University School of Medicine, Shanghai 200092, China

^2^ Department of Clinical Nutrition, College of Health Science and Technology, Shanghai Jiao Tong University School of Medicine, Shanghai 200135, China

^3^ Medical Food Laboratory, Shanghai Institute for Pediatric Research, Shanghai 200092, China

^4^ Shanghai Pharma Qingchunbao-Xinhua Hospital Precision Nutrition Research Center, Chiatai Qingchunbao Pharmaceutical Group Limited, Hangzhou 310001, Zhejiang, China

^*^Corresponding authors at:

Shanghai Key Laboratory of Pediatric Gastroenterology and Nutrition, Xinhua Hospital, Shanghai Jiao Tong University School of Medicine, Shanghai 200092, China. E-mail: jzhong@shsmu.edu.cn (J. Zhong), caiw1978@163.com (W. Cai)

Abbreviated running title: Preparation of low-protein cookies

**Table S1.** Comparisons of the detected volatile organic compounds (VOCs) in the special low protein cookies (SLPC, n=3) with different baking temperatures by headspace-gas chromatography-mass spectrometry (HS-GC-IMS).

| Volatile compounds | CAS# | Formula | Molecular  weight | Retention  index | Retention time  [sec] | Drift time [RIP  relative] | Peak Volume (a.u.) | | | | | |  |
| --- | --- | --- | --- | --- | --- | --- | --- | --- | --- | --- | --- | --- | --- |
|  |  |  |  |  |  |  | **100℃** | **120℃** | **140℃** | **160℃** | **180℃** | **200℃** |  |
|  |  |  |  |  |  |  |  |  |  |  |  |  |  |
| **Alcohols** | | | | | | | | | | | | |  |
| 2-Propanol-M | 67-63-0 | C3H8O | 60.1 | 948.6 | 273.412 | 1.0908 | 386.46±4.59 | 339.94±7.05 | 295.37±9.51 | 299.95±5.62 | 316.89±8.59 | 239.51±4.18 |  |
| 2-Propanol-D | 67-63-0 | C3H8O | 60.1 | 948.9 | 273.746 | 1.2168 | 274.69±8.56 | 158.71±3.70 | 98.44±14.45 | 103.02±7.96 | 112.79±4.26 | 64.17±3.49 |  |
| ethanol-M | 64-17-5 | C2H6O | 46.1 | 954.3 | 278.841 | 1.04101 | 1620.93±28.93 | 1633.53±4.74 | 1059.92±4.72 | 1024.49±44.64 | 997.85±8.81 | 999.35±35.46 |  |
| ethanol-D | 64-17-5 | C2H6O | 46.1 | 955.7 | 280.159 | 1.13106 | 1885.45±15.10 | 1933.40±28.88 | 1084.16±30.61 | 907.58±77.22 | 849.29±10.39 | 838.29±31.82 |  |
| 1-propanol | 71-23-8 | C3H8O | 60.1 | 1063.6 | 413.716 | 1.1087 | 387.39±11.91 | 363.92±3.59 | 139.37±6.98 | 100.27±2.62 | 199.38±14.65 | 316.65±7.05 |  |
| Butanol-M | 71-36-3 | C4H10O | 74.1 | 1167.6 | 595.743 | 1.18157 | 72.97±5.06 | 72.67±4.09 | 64.36±1.65 | 162.28±5.86 | 320.77±5.84 | 363.61±8.42 |  |
| Butanol-D | 71-36-3 | C4H10O | 74.1 | 1167.9 | 596.281 | 1.38008 | 41.82±3.55 | 43.81±5.29 | 43.77±3.62 | 60.89±3.90 | 145.06±4.63 | 193.26±8.59 |  |
| 1-Penten-3-ol | 616-25-1 | C5H10O | 86.1 | 1181.1 | 623.753 | 0.94161 | 216.95±8.19 | 239.63±6.65 | 207.99±2.00 | 223.32±4.97 | 345.91±5.16 | 317.48±15.78 |  |
| 1-Pentanol-M | 71-41-0 | C5H12O | 88.1 | 1274 | 802.202 | 1.25268 | 302.34±7.25 | 521.92±13.18 | 426.28±20.32 | 503.39±8.37 | 999.89±3.46 | 928.52±3.83 |  |
| 1-Pentanol-D | 71-41-0 | C5H12O | 88.1 | 1273.6 | 801.219 | 1.5116 | 222.31±16.64 | 380.51±6.91 | 271.73±3.59 | 358.86±15.94 | 1230.72±51.12 | 1273.14±15.25 |  |
| (E) -3-hexen-1-ol | 928-97-2 | C6H12O | 100.2 | 1346.1 | 944.565 | 1.24109 | 756.99±37.81 | 868.95±31.13 | 845.15±2.62 | 1004.96±81.53 | 1256.99±55.69 | 1378.01±21.18 |  |
| 1-Hexanol | 111-27-3 | C6H14O | 102.2 | 1374.8 | 1003.798 | 1.32643 | 190.79±1.24 | 195.64±1.86 | 115.15±1.17 | 137.49±3.76 | 177.16±5.00 | 258.01±9.01 |  |
| (E)-2-Hexen-1-ol | 928-95-0 | C6H12O | 100.2 | 1419.2 | 1103 | 1.34274 | 366.68±31.46 | 433.47±26.90 | 452.63±13.76 | 533.78±91.95 | 518.16±21.23 | 752.59±36.61 |  |
| 1-Octen-3-ol | 3391-86-4 | C8H16O | 128.2 | 1476 | 1244.154 | 1.15682 | 237.80±24.23 | 241.51±10.66 | 257.07±10.76 | 233.22±29.19 | 239.09±4.27 | 248.32±9.17 |  |
| **Aldehydes** | | | | | | | | | | | | |  |
| Propionaldehyde-M | 123-38-6 | C3H6O | 58.1 | 829.2 | 181.354 | 1.07701 | 405.44±8.34 | 506.56±2.30 | 455.21±1.11 | 395.96±2.86 | 544.39±18.38 | 621.32±10.78 |  |
| Propionaldehyde-D | 123-38-6 | C3H6O | 58.1 | 830.8 | 182.339 | 1.14067 | 210.22±22.82 | 419.60±6.72 | 300.01±5.40 | 196.04±2.20 | 505.07±55.28 | 870.01±41.20 |  |
| methylpropanal | 78-84-2 | C4H8O | 72.1 | 846.1 | 192.19 | 1.11504 | 2238.96±40.09 | 1475.95±22.67 | 1472.63±43.62 | 778.37±43.96 | 764.37±28.07 | 1121.01±12.48 |  |
| acrolein | 107-02-8 | C3H4O | 56.1 | 866.2 | 205.98 | 1.05634 | 632.92±16.93 | 213.74±9.95 | 211.40±4.60 | 147.85±4.74 | 242.32±54.85 | 413.31±11.62 |  |
| Butanal-M | 123-72-8 | C4H8O | 72.1 | 891.9 | 225.024 | 1.13075 | 205.40±15.16 | 280.37±5.66 | 228.71±6.58 | 266.21±13.53 | 407.05±19.72 | 500.50±9.28 |  |
| Butanal-D | 123-72-8 | C4H8O | 72.1 | 892.4 | 225.353 | 1.27955 | 65.57±2.87 | 114.14±5.19 | 87.33±8.41 | 106.21±12.42 | 239.58±33.79 | 449.38±28.45 |  |
| Pentanal-D | 110-62-3 | C5H10O | 86.1 | 996.8 | 322.763 | 1.41797 | 148.92±2.02 | 671.13±4.11 | 534.71±13.76 | 566.70±9.94 | 1549.17±179.40 | 1692.68±35.52 |  |
| Pentanal-M | 110-62-3 | C5H10O | 86.1 | 997.4 | 323.475 | 1.19447 | 284.34±9.88 | 644.86±15.49 | 650.64±9.06 | 634.89±10.55 | 855.42±11.08 | 865.93±27.74 |  |
| Hexanal-M | 66-25-1 | C6H12O | 100.2 | 1097.4 | 469.165 | 1.26839 | 826.93±58.37 | 1052.81±38.68 | 1018.36±44.66 | 919.76±48.44 | 2079.06±78.51 | 2881.07±51.58 |  |
| Hexanal-D | 66-25-1 | C6H12O | 100.2 | 1097.8 | 469.79 | 1.56018 | 156.17±11.89 | 199.17±7.20 | 154.86±4.91 | 171.00±8.36 | 574.50±70.67 | 1637.83±54.06 |  |
| (E)-2-Pentenal-M | 1576-87-0 | C5H8O | 84.1 | 1144.2 | 550.026 | 1.1051 | 114.63±0.94 | 163.55±2.57 | 166.57±7.61 | 162.58±2.61 | 262.94±25.99 | 296.87±15.14 |  |
| (E)-2-Pentenal-D | 1576-87-0 | C5H8O | 84.1 | 1144.4 | 550.564 | 1.35656 | 57.81±3.28 | 59.35±2.51 | 62.26±2.50 | 55.24±0.78 | 106.93±8.74 | 146.76±9.65 |  |
| Heptaldehyde-M | 111-71-7 | C7H14O | 114.2 | 1198.9 | 659.12 | 1.33825 | 92.14±5.35 | 128.85±5.69 | 154.17±5.08 | 185.96±13.64 | 343.63±13.69 | 595.95±7.62 |  |
| Heptaldehyde-D | 111-71-7 | C7H14O | 114.2 | 1198.9 | 659.12 | 1.68905 | 75.97±10.10 | 79.08±8.74 | 78.40±2.99 | 70.82±2.89 | 132.22±8.99 | 410.25±14.47 |  |
| (E)-2-hexenal-M | 6728-26-3 | C6H10O | 98.1 | 1233.6 | 721.624 | 1.17683 | 160.26±4.66 | 238.35±24.45 | 216.93±18.54 | 253.59±16.00 | 627.25±6.69 | 655.21±15.76 |  |
| (E)-2-hexenal-D | 6728-26-3 | C6H10O | 98.1 | 1232.8 | 720.15 | 1.5116 | 265.43±13.40 | 143.94±10.48 | 128.25±13.85 | 141.38±0.21 | 534.58±17.49 | 729.68±25.61 |  |
| (E)-2-Heptenal-M | 18829-55-5 | C7H12O | 112.2 | 1338.2 | 928.878 | 1.25503 | 211.83±16.30 | 369.58±6.57 | 387.90±20.27 | 522.55±3.03 | 1091.64±21.98 | 1087.63±40.94 |  |
| (E)-2-Heptenal-D | 18829-55-5 | C7H12O | 112.2 | 1337.9 | 928.212 | 1.66452 | 210.84±26.14 | 357.18±3.07 | 311.36±2.27 | 451.90±21.15 | 1889.49±51.15 | 3040.75±21.79 |  |
| octanal-M | 124-13-0 | C8H16O | 128.2 | 1303.2 | 862.258 | 1.40572 | 97.64±11.83 | 91.41±13.48 | 99.93±8.16 | 118.66±4.22 | 200.18±13.23 | 397.72±6.22 |  |
| octanal-D | 124-13-0 | C8H16O | 128.2 | 1303.5 | 862.924 | 1.81522 | 58.15±5.65 | 55.02±2.41 | 61.30±4.35 | 54.11±2.36 | 74.88±6.49 | 189.94±3.98 |  |
| ( E)-2-octenal-M | 2548-87-0 | C8H14O | 126.2 | 1431 | 1130.901 | 1.33288 | 98.57±14.42 | 126.58±3.70 | 120.55±4.13 | 130.58±4.64 | 320.26±5.34 | 342.82±1.75 |  |
| ( E)-2-octenal-D | 2548-87-0 | C8H14O | 126.2 | 1431.4 | 1131.915 | 1.81485 | 173.12±4.80 | 172.35±4.26 | 158.28±11.85 | 165.86±13.45 | 324.32±23.00 | 915.60±11.99 |  |
| furfural-D | 98-01-1 | C5H4O2 | 96.1 | 1458.7 | 1199.385 | 1.33147 | 696.21±40.89 | 750.63±31.61 | 996.39±83.56 | 1034.53±44.32 | 1525.44±18.88 | 7827.82±115.74 |  |
| furfural-M | 98-01-1 | C5H4O2 | 96.1 | 1462.9 | 1210.038 | 1.08343 | 595.85±58.78 | 736.14±4.33 | 948.55±77.77 | 872.52±59.20 | 1107.32±41.97 | 2295.92±55.78 |  |
| ( E, E)-2,4-heptadienal-D | 4313-03-5 | C7H10O | 110.2 | 1478.4 | 1250.59 | 1.61522 | 175.26±15.91 | 166.20±5.35 | 170.29±20.25 | 166.36±6.28 | 268.75±2.73 | 447.38±10.88 |  |
| ( E, E)-2,4-heptadienal-M | 4313-03-5 | C7H10O | 110.2 | 1478.6 | 1251.175 | 1.19681 | 72.29±8.28 | 85.19±1.80 | 87.22±6.02 | 99.51±4.60 | 326.14±6.20 | 271.97±6.83 |  |
| 5-Methylfurfural-M | 620-02-0 | C6H6O2 | 110.1 | 1531 | 1398.218 | 1.13279 | 212.97±10.37 | 207.50±20.96 | 215.78±12.12 | 211.74±15.74 | 261.85±15.88 | 1200.01±12.07 |  |
| 5-Methylfurfural-D | 620-02-0 | C6H6O2 | 110.1 | 1530.7 | 1397.341 | 1.47383 | 265.14±19.64 | 274.47±22.49 | 301.53±12.37 | 289.96±2.43 | 334.75±8.83 | 1673.31±7.78 |  |
| nonanal-M | 124-19-6 | C9H18O | 142.2 | 1404.6 | 1069.302 | 1.48118 | 408.81±41.71 | 496.08±29.69 | 472.56±13.23 | 520.41±18.87 | 970.55±19.23 | 1076.32±10.18 |  |
| nonanal-D | 124-19-6 | C9H18O | 142.2 | 1403.8 | 1067.552 | 1.93439 | 134.28±8.37 | 158.85±9.18 | 160.36±8.90 | 176.89±4.66 | 440.71±33.00 | 586.99±3.55 |  |
| **Ketones** | | | | | | | | | | | | |  |
| 2-Butanone-M | 78-93-3 | C4H8O | 72.1 | 914 | 242.755 | 1.06296 | 385.05±2.68 | 309.11±1.17 | 306.28±9.49 | 134.74±2.91 | 122.75±0.72 | 206.26±6.18 |  |
| 2-Butanone-D | 78-93-3 | C4H8O | 72.1 | 913.2 | 242.098 | 1.244 | 684.60±26.97 | 320.51±6.14 | 296.11±26.00 | 65.14±0.97 | 66.17±2.77 | 163.76±4.51 |  |
| 2,3 Butanedione | 431-03-8 | C4H6O2 | 86.1 | 960.6 | 284.962 | 1.18246 | 794.59±16.75 | 793.85±34.37 | 744.10±16.70 | 587.33±77.12 | 505.20±22.55 | 540.11±46.28 |  |
| 2-Pentanone-M | 107-87-9 | C5H10O | 86.1 | 992.7 | 318.259 | 1.11855 | 486.97±26.82 | 110.34±6.86 | 124.73±7.29 | 87.83±6.95 | 90.58±9.67 | 100.73±7.52 |  |
| 2-Pentanone-D | 107-87-9 | C5H10O | 86.1 | 993.7 | 319.352 | 1.36763 | 850.37±43.43 | 115.60±1.21 | 123.25±7.52 | 91.85±1.38 | 133.93±9.81 | 199.15±8.74 |  |
| 2-Hexanone | 591-78-6 | C6H12O | 100.2 | 1096.3 | 467.293 | 1.51993 | 62.98±2.07 | 44.73±2.00 | 41.60±2.38 | 39.10±1.48 | 80.19±5.55 | 179.10±2.27 |  |
| 2-Heptanone-M | 110-43-0 | C7H14O | 114.2 | 1195.9 | 653.963 | 1.26082 | 296.38±15.74 | 125.18±5.67 | 107.21±4.67 | 106.38±5.38 | 145.66±6.35 | 196.42±5.59 |  |
| 2-Heptanone-D | 110-43-0 | C7H14O | 114.2 | 1195.3 | 652.837 | 1.62406 | 104.47±2.45 | 40.57±1.68 | 39.42±2.61 | 39.11±2.42 | 60.13±4.93 | 130.37±2.22 |  |
| 2-Methyltetrahydrofuran-3-one-M | 3188-00-9 | C5H8O2 | 100.1 | 1278.4 | 811.537 | 1.0696 | 78.84±7.96 | 69.05±4.22 | 69.57±6.89 | 73.62±3.72 | 74.60±3.32 | 321.06±4.93 |  |
| 2-Methyltetrahydrofuran-3-one-D | 3188-00-9 | C5H8O2 | 100.1 | 1278.7 | 812.029 | 1.41745 | 61.50±4.14 | 59.43±1.40 | 75.52±4.65 | 68.76±3.06 | 107.14±2.77 | 387.53±2.86 |  |
| 3-Hydroxy-2-butanone-M | 513-86-0 | C4H8O2 | 88.1 | 1298 | 852.931 | 1.06502 | 244.90±44.25 | 386.12±8.19 | 402.08±11.98 | 260.51±8.85 | 236.82±15.41 | 400.59±10.38 |  |
| 3-Hydroxy-2-butanone-D | 513-86-0 | C4H8O2 | 88.1 | 1298 | 852.931 | 1.32546 | 183.21±32.91 | 243.25±2.97 | 249.00±11.15 | 161.95±4.71 | 154.11±12.72 | 388.96±8.28 |  |
| Cyclohexanone-M | 108-94-1 | C6H10O | 98.1 | 1301 | 858.261 | 1.15184 | 498.99±29.01 | 364.91±3.48 | 340.64±10.02 | 439.49±16.38 | 422.50±13.39 | 287.79±7.70 |  |
| Cyclohexanone-D | 108-94-1 | C6H10O | 98.1 | 1299.9 | 856.263 | 1.44995 | 540.48±17.65 | 536.62±12.81 | 517.43±20.23 | 453.55±34.94 | 392.86±23.10 | 406.16±19.24 |  |
| **Esters** | | | | | | | | | | | | | |
| methyl acetate-M | 79-20-9 | C3H6O2 | 74.1 | 856.3 | 199.085 | 1.03072 | 40.40±4.13 | 70.95±3.14 | 78.72±3.55 | 63.99±2.16 | 81.16±1.50 | 225.07±10.20 |  |
| methyl acetate-D | 79-20-9 | C3H6O2 | 74.1 | 855.9 | 198.757 | 1.19027 | 33.92±0.98 | 35.25±1.42 | 38.68±8.24 | 33.81±4.04 | 36.73±4.55 | 98.66±5.99 |  |
| Ethyl Acetate | 141-78-6 | C4H8O2 | 88.1 | 902.8 | 233.561 | 1.09437 | 55.51±4.31 | 113.12±3.29 | 113.81±6.80 | 77.74±1.37 | 73.57±1.66 | 112.49±5.69 |  |
| butyl acetate | 123-86-4 | C6H12O2 | 116.2 | 1078.1 | 436.712 | 1.23444 | 53.37±6.05 | 108.94±7.15 | 129.15±0.72 | 95.18±3.52 | 92.97±3.31 | 267.29±7.24 |  |
| pentyl acetate | 628-63-7 | C7H14O2 | 130.2 | 1188.7 | 639.963 | 1.3098 | 61.36±1.63 | 62.67±3.37 | 57.10±3.75 | 59.57±3.28 | 147.00±3.12 | 197.58±6.29 |  |
| ethyl 2-hydroxypropanoate | 97-64-3 | C5H10O3 | 118.1 | 1312.9 | 880.24 | 1.14523 | 107.36±8.69 | 310.30±3.38 | 477.26±17.02 | 463.88±13.94 | 285.17±8.27 | 436.89±4.64 |  |
| Citronellyl formate-M | 105-85-1 | C11H20O2 | 184.3 | 1655.7 | 1821.609 | 1.42509 | 895.16±54.66 | 824.50±28.58 | 785.59±32.88 | 880.75±40.73 | 1469.30±36.42 | 2890.94±76.38 |  |
| Citronellyl formate-D | 105-85-1 | C11H20O2 | 184.3 | 1655.9 | 1822.654 | 2.08727 | 353.40±38.33 | 326.16±11.37 | 344.66±7.22 | 313.70±20.90 | 370.36±15.09 | 1406.97±66.24 |  |
| **Acids** | | | | | | | | | | | | |  |
| Acetic acid-M | 64-19-7 | C2H4O2 | 60.1 | 1451.6 | 1181.402 | 1.06622 | 1978.10±84.05 | 2134.70±66.85 | 2303.36±31.24 | 2387.31±35.98 | 2241.73±31.46 | 2378.56±44.99 |  |
| Acetic acid-D | 64-19-7 | C2H4O2 | 60.1 | 1451.8 | 1182.063 | 1.16124 | 1116.69±89.50 | 1114.41±67.66 | 1356.57±30.10 | 1369.69±51.99 | 1553.41±45.41 | 2155.50±57.69 |  |
| Propionic acid-M | 79-09-4 | C3H6O2 | 74.1 | 1513.9 | 1348.376 | 1.11163 | 908.12±15.87 | 1008.61±63.83 | 1155.05±38.46 | 1009.12±38.75 | 903.60±14.05 | 1189.87±35.90 |  |
| Propionic acid-D | 79-09-4 | C3H6O2 | 74.1 | 1513.1 | 1346.132 | 1.26507 | 301.50±15.37 | 379.22±37.35 | 453.85±12.65 | 377.67±17.38 | 272.38±10.34 | 551.99±45.57 |  |
| **Furan** | | | | | | | | | | | | |  |
| 2-pentylfuran | 3777-69-3 | C9H14O | 138.2 | 1248.4 | 750.121 | 1.24614 | 145.82±0.41 | 131.51±5.77 | 136.61±4.12 | 155.25±6.40 | 426.60±23.50 | 1009.17±16.14 |  |
| 2-acetylfuran-M | 1192-62-7 | C6H6O2 | 110.1 | 1487.8 | 1275.817 | 1.12226 | 138.82±2.10 | 150.31±6.84 | 167.74±17.24 | 163.16±8.15 | 216.88±4.10 | 723.07±13.15 |  |
| 2-acetylfuran-D | 1192-62-7 | C6H6O2 | 110.1 | 1487.8 | 1275.817 | 1.43826 | 426.65±174.52 | 244.09±23.80 | 242.37±24.90 | 214.36±13.11 | 215.90±13.31 | 1558.65±25.23 |  |
| Benzene | | | | | | | | | | | | |  |
| Propyl-benzene | 103-65-1 | C9H12 | 120.2 | 1215.7 | 688.704 | 1.17814 | 45.63±8.49 | 43.33±0.87 | 45.46±1.28 | 42.88±4.61 | 87.48±7.56 | 181.14±8.81 |  |

**
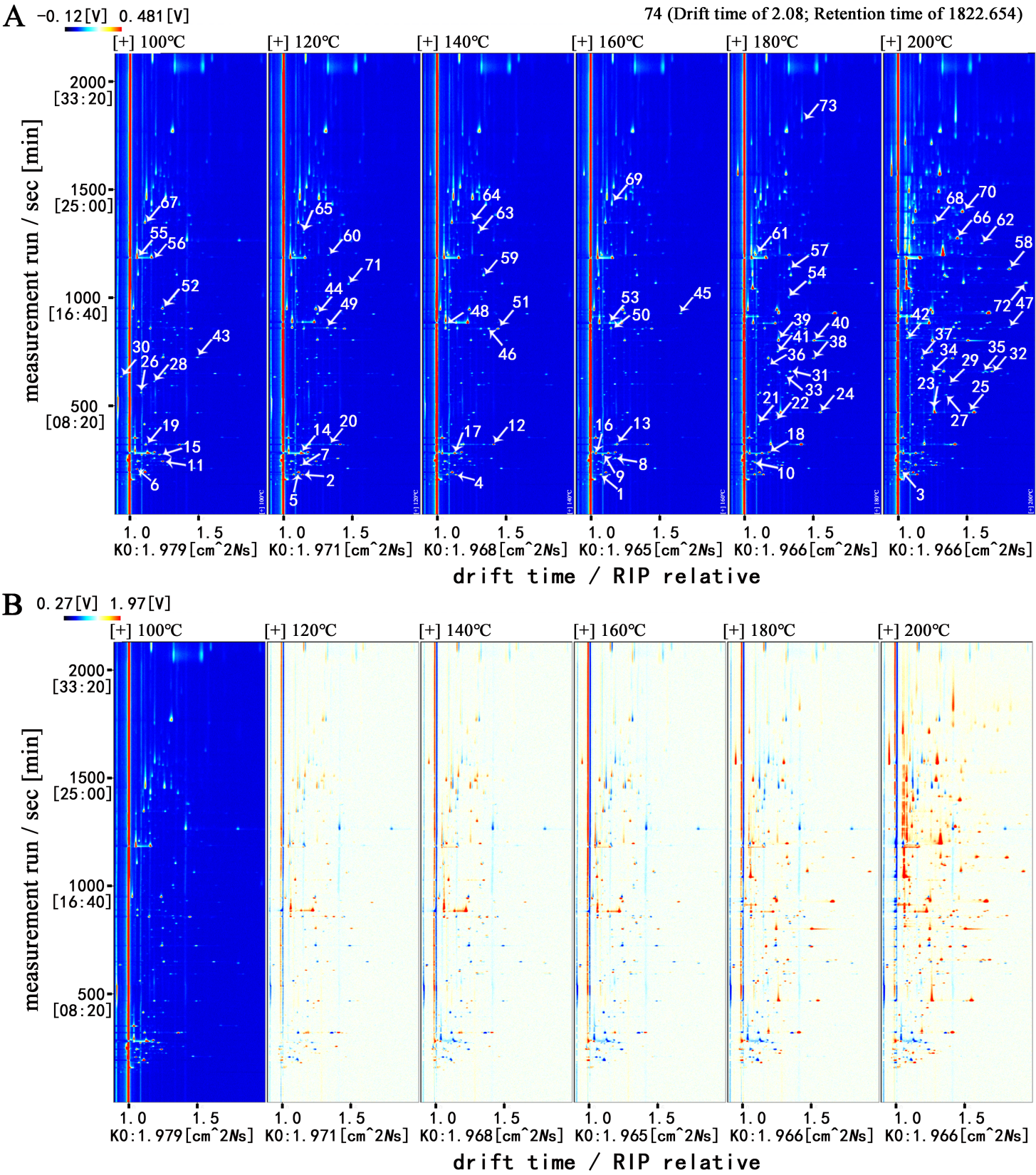
**

**Fig. S1.** Volatile fingerprints of the LPCs with different baking temperatures using headspace-gas chromatography-ion mobility spectrometry (HS-GC-IMS). (A): Topographic plots. Numbers (1–73) indicate the areas (spots, peaks) that are the identified volatile organic compounds (VOCs) characterized by the retention time (on the y-axis), the relative drift time (on the x-axis), and the intensity of the signals. The redder the area is, the larger the quantity of VOC is. The information on the number 74 was written in the top-right corner. (B): Topographic subtraction plots. They were obtained by subtracting the SLPC plot with 100°C (The leftmost image) from the other sample plots. In the topographic subtraction plots (The 5 images on the right), the red and blue colors indicate more or less, respectively, contents than the SLPC plot (The leftmost image) with 100°C.
